# An odorant-binding protein functions in fire ant social immunity interfacing with innate immunity

**DOI:** 10.1098/rsob.240254

**Published:** 2025-02-12

**Authors:** Wei Zhang, Xuanyu Chen, Jiaxin Tian, Coby Schal, Amr Mohamed, Lian-Sheng Zang, Yuxian Xia, Nemat O. Keyhani

**Affiliations:** ^1^ State Key Laboratory of Green Pesticide, Key Laboratory of Green Pesticide and Agricultural Bioengineering (Ministry of Education), Guizhou University, Guiyang, Huaxi District 550025, People’s Republic of China; ^2^ Department of Biological Sciences, University of Illinois, Chicago, IL 60607, USA; ^3^ School of Life Science, Chongqing University, Chongqing 401331, People’s Republic of China; ^4^ Department of Entomology and Plant Pathology, North Carolina State University, Raleigh, NC, USA; ^5^ Department of Entomology, Faculty of Science, Cairo University, Giza 12613, Egypt

**Keywords:** odorant-binding protein, sanitation behaviour, immunity and olfaction, *Solenopsis invicta*, apolipophorin-III, fatty acid binding protein

## Abstract

Social immunity-mediated sanitation behaviours occur in insects when microbially killed corpses are removed and/or dismembered by healthy nestmates. However, little is known concerning the chemical signals or receptor proteins that mediate these responses. Here, we identify cuticular components in the eusocial red important fire ant, *Solenopsis invicta*: behenic acid, which induces dismemberment behaviour, and oleic and *cis*,*cis*-9,12-linoleic acids, which inhibit dismemberment in a process mediated by *S. invicta* odorant-binding protein-15 (SiOBP15). Yeast two-hybrid screening and protein–protein interaction analyses identified the ant immunity-related proteins apolipophorin-III (SiApoLp-III) and fatty acid binding protein-5 (SiFABP5) as SiOBP15 interacting partners. SiOBP15 and SiFABP5 bound all three dismemberment-related compounds, whereas interactions between SiOBP15 and SiApoLp-III narrowed binding to behenic acid. RNAi-mediated gene expression knockdown of *SiOBP15*, *SiApoLp-III* or *SiFABP5* revealed that behenic acid chemoreception determines dismemberment behaviour via SiApoLp-III/SiOBP15, whereas SiOBP15 or SiOBP15/SiFABP5 recognition of linoleic acid inhibits dismemberment behaviour. These data identify a host circuit linking olfactory proteins and proteins involved in innate immunity to control the degree of sanitation behaviour elicited in response to microbial infection. We identify specific chemical cues transduced by these proteins, providing a mechanism connecting olfaction-related processes to innate immunity, host–pathogen interactions and social immunity.

**Figure:**
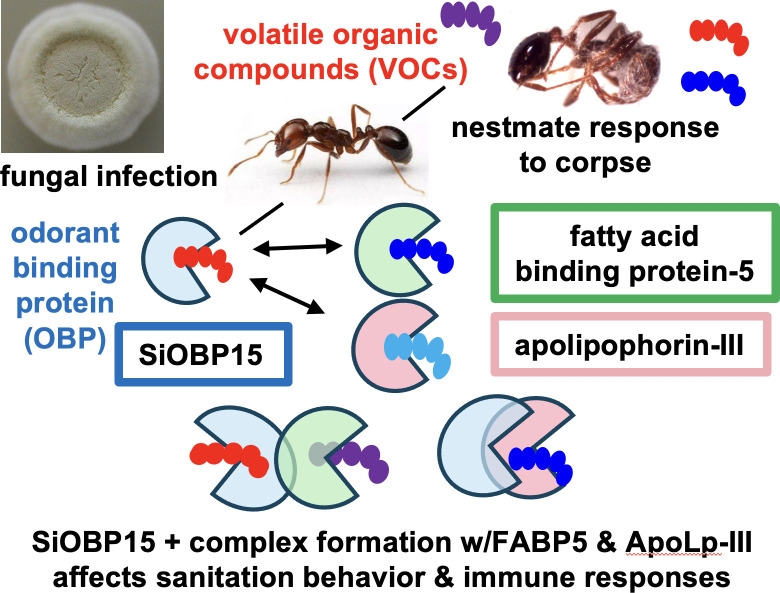

## 1. Background

The emergence of sociality in insects represents an important evolutionary step, with eusocial insects considered the pinnacle of sociality [1–3]. However, because social insect colonies often consist of high densities of thousands to even millions of individuals living in a microbe-rich soil environment often at high relative humidity and temperature, they are exposed to a wide range of microbial pathogens [4,5]. All insects possess innate immunity mechanisms that defend against microbial pathogens [6], but social insects have evolved additional layers of defences against microbial pathogens that arise from interactions with conspecifics within the shared nest environment. These behavioural adaptations have been placed under the umbrella term ‘social immunity’ [7–9] and include grooming, trophallaxis (transfer of food or other fluids between nestmates) to mitigate community-wide pathogen infections and even aggression (towards diseased individuals) [10]. In addition, many eusocial insects, particularly ant species (e.g. the red imported fire ant, *Solenopsis invicta*), display specific social immunity based ‘sanitation’ behaviours. These behaviours are exhibited by healthy individuals towards dead nestmates and include corpse removal (necrophoretic behaviour), burial or placement of corpses in ‘cemeteries’ or ‘bone piles’ away from the nest, and/or dismemberment of corpses [10–12]. Social immune mechanisms such as sanitation of infected corpses act to decrease the spread of infectious agents and require some form of interaction or communication between healthy and infected/dead organisms [13].

Insect olfaction is exquisitely sensitive, and the production and perception of environmental chemicals including semiochemicals, volatile compounds, hydrocarbons and fatty acids, pheromones and other compounds and metabolites are well-known drivers of insect behaviours [14–17]. Insects possess specialized structures (sensilla) found on the antennae and other body parts that contain two to three sensory neurons, which in turn house the olfactory receptors (ORs) [18]. Environmental compounds enter the olfactory sensillar lymph fluid via pores and are thought to be recognized by two classes of small molecular weight (10–20 kDa) soluble factors known as chemosensory proteins and olfactory binding proteins (CSPs and OBPs, respectively) that shuttle these compounds to the ORs [19]. Both CSPs and OBPs exist as extended gene families in almost all insects, and recent evidence suggests that specific CSP/OBP members function in diverse physiological processes beyond canonical olfactory pathways [20–23]. The characterization and functional roles of several OBPs in non-social insect chemical perception and behavioural regulation have been reported [24–26]. In *S. invicta*, SiOBP3 (originally named gp-9) has been linked to colony social organization [27]. In honey bees, OBP16 and OBP18 were found highly expressed in the antennae of bees engaging in high levels of hygiene behaviour, including corpse removal [28]. CSP/OBPs are also involved in detection of microbial pathogens; for example, in locusts (*Locusta migratoria*), LmigCSP60 was found responsible for detecting and mediating avoidance behaviour to volatiles in fungal contaminated food [29]. In addition, LmigOBP11 was found to also respond to fungal volatiles, but in this case, the protein was identified as suppressing locust innate immune responses (i.e. *Toll*-pathway mediated responses to fungal infection) [20]. However, whether any direct molecular connections between olfaction and innate immunity exist remains unknown, much less whether such interactions can impact social immunity behaviours [30].

*Solenopsis invicta* is one of the world’s most successful invasive species, having spread from northern Argentina and southern Brazil to the United States, and subsequently worldwide [31,32]. With the characterization of a ‘social mini-chromosome’ implicated in mediating colony organization (mono- versus polygyne colony structure) [33], this ant species has emerged as a model system for critical aspects of social behaviour [34]. Entomopathogenic fungi, including broad host range members of the *Beauveria* and *Metarhizium* genera, can readily kill fire ants under laboratory conditions [35]; however, their effectiveness is significantly lower in the field [4]. Social immunity-mediated sanitation behaviours are considered prime factors that restrict the efficacy of these fungal pesticides, and both corpse removal and corpse-dismemberment occur in *S. invicta* [36]. Volatile substances (e.g. fatty acids) produced by the insect pathogenic fungus *Metarhizium anisopliae* on infected fire ant pupae elicit corpse removal behaviour [37], and phenyl acetate, 2-phenylethanol and benzyl alcohol released from fungal-infected bees also stimulate hygiene behaviour [38]. Compounds such as octanol and octanone, released from normal dead termites, stimulate corpse-dismemberment behaviour in healthy termites [39]. However, despite the central nature of sanitation and related behaviours in microbial disease and immunity responses, information concerning the chemical signals produced due to mortality from microbial infection and how these signals are recognized by healthy conspecifics to determine decisions on whether to engage in corpse-dismemberment behaviour towards the dead nestmate remains almost completely lacking.

Here, we define the chemical signals and protein receptors involved in connecting olfaction to innate immunity to the dismemberment behaviour seen as part of social immunity responses. Our data show that fire ants infected and killed by the generalist fungal insect pathogen *Beauveria bassiana* are dismembered by healthy nestmates at significantly higher levels than freeze-killed workers. Three volatile chemicals from *B. bassiana*-infected corpses were identified, with behenic acid shown to induce increased corpse-dismemberment behaviour, whereas *cis*,*cis*-9-12-linoleic acid and oleic acid inhibited corpse-dismemberment behaviour. RNAi-mediated knockdown of *S. invicta* odorant-binding protein-15 (*SiOBP15)* expression significantly decreased corpse-dismemberment behaviour towards freeze-killed ants but not towards *B. bassiana*-killed ants. We show that SiOBP15 has broad substrate specificity and is capable of binding all three fatty acids. We further identify two protein interacting partners of SiOBP15, the innate immunity-related *S. invicta* apolipophorin-III (SiApoLp-III) and *S. invicta* fatty acid binding protein-5 (SiFABP5), which were isolated via yeast two-hybrid screening, with each respective protein-protein interaction confirmed by demonstrating direct binding *in vitro*. Interactions between the proteins resulted in altered ligand-binding specificities. RNAi knockdown of *SiApoLp-III* and *SiFABP5* expression resulted in altered corpse-dismemberment behaviour towards freeze-killed and *B. bassiana*-killed ants. These results define a network linking olfaction (SiOBP15) to innate immunity (SiApolp-III/SiFABP5) in the regulation of the degree to which workers engage in dismemberment behaviour (social immunity).

## 2. Material and methods

### 2.1. Insects and fungal cultures

Red imported fire ants (*S. invicta*) were collected in Guangzhou, China, in 2019, and determined to be polygyne by queen numbers and PCR verification of the *gp-9* allele sequence and used to establish an in-house colony. The colony was kept in plastic boxes with talcum powder dusted on the inner side walls at 26°C and approximately 70% humidity and under a 16:8 light:dark photoperiod. Sucrose was available as a carbon source, and periodically cricket or cockroach cadavers were added to the colony. Fungal cultures of *B. bassiana* (ATCC 90517) were cultured on potato dextrose agar for 15 days at 26°C. Conidial suspensions were prepared by harvesting cells from plates in sterile 0.05% Tween-80; hyphae/mycelia were removed from the suspension by filtering through sterile lens paper, and conidial concentrations were determined by counting using a hemocytometer. Harvested conidia were adjusted to the desired concentration in sterile 0.05% Tween-80.

### 2.2. Dismemberment behavioural assay

Fresh corpses of workers killed by either rapid immersion in liquid nitrogen were collected and maintained at −80°C until use. Infection was performed by immersing workers for 5 s in 1 × 10^8^ suspensions of fungal conidia. Infected workers were kept in isolated boxes until mortality (3–4 days) and were then used immediately for dismemberment assays. Each technical replicate of a dismemberment assay used five workers selected from different treatment groups (i.e. killed by freezing, killed by *B. bassiana* or subjected to another treatment as indicated). Of the five ‘killed’ workers, one was placed near the edge of the container in a ‘+’ pattern of a plastic container (9 cm diameter) and one in the centre. To each container was added 15 healthy workers or 15 workers treated as described below (e.g. RNAi treated). Containers contained an Eppendorf tube with a sterile solution of 5% sucrose. Corpse-dismemberment behaviour was assessed every 12 h for three consecutive days, and corpse limbs or other parts that were dismembered were counted. The corpse-dismemberment percentage was calculated by dividing the total number of dismembered parts (5 corpses × 10 body parts/each cage/3 days). Each experiment contained three technical replicates, and the entire experiment was repeated at least three times.

### 2.3. Chemical analyses

Cuticular chemicals derived from *B. bassiana*-killed workers and workers killed by freezing were analysed using a gas chromatograph system coupled to a mass spectrometer (GC–MS) as described [40] with minor modifications. For fatty acids analyses, 15 *B. bassiana*-killed workers or 15 workers killed by freezing were immersed in 0.3 ml of methylene chloride for 10 min for chemical extraction. After centrifuged at 3000*g* for 5 min at 4°C, 50 μl supernatant was transferred to a 1.5 ml Eppendorf tube and dried down completely in a vacuum concentrator without heating. To the resultant pellet, 200 μl methanol was added to dissolve the residue, after which 40 μl of the methylation agent (trimethylsilyl) diazomethane was added, followed by drying using nitrogen flow, after which 100 μl hexane was added to the dried material, and 90 μl of the solution was analysed by GC–MS. GC–MS analysis was performed using a Shimadzu 2010 Plus. In total, 51 fatty acid standards were used to quantify respective concentrations in freeze-killed and *B. bassiana*-killed workers. The system consisted of a Thermo capillary column, with 1 μl aliquot of the analyte injected in split mode (5:1). Helium was used as the carrier gas, the inlet purge flow was 5 ml min^−1^, and the gas flow rate through the column was 0.88 ml min^−1^. The initial temperature was 50°C hold for 1 min; raised to 175°C at 20°C min^−1^, hold for 0 min; raised to 190°C at 2°C min^−1^, hold on 5 min; raised to 225°C at 5°C min^−1^, hold for 0 min; raised to 240°C at 10°C min^−1^, hold for 1 min. The injection, transfer line and ion source temperatures were 240, 240 and 200°C, respectively. The mass spectrometry data were acquired in scan mode after solvent delay of 4 min. The entire experiment was repeated twice.

For volatile organic compounds (VOCs) analysis, 50 workers were placed into a 20 ml headspace bottle, and 10 μl of 2-octanol was added (10 mg l^−1^ stock in dH_2_O) as internal standard. All samples were analysed by GC–MS. For solid-phase microextraction (SPME) on the PAL rail system (Zwingen, Switzerland), incubation temperature was 60°C, preheat time was 15 min, incubation time was 30 min and desorption time was 4 min. GC–MS analysis was performed using an Agilent 7890 GC coupled with a 5977B MS. The system utilized a DB-Wax column. Injections were in splitless mode with helium as the carrier gas at a flow rate of 1 ml min^−1^ and the front inlet purge flow was 3 ml min^−1^. The initial temperature was kept at 40°C for 4 min, then raised to 245°C (5°C per min) and maintained for 5 min. The injection, transfer line, ion source and quad temperatures were 250, 250, 230 and 150°C, respectively. The energy was −70 eV in electron impact mode. The MS data were acquired in scan mode with the m/z range of 20−500 and no solvent delay.

### 2.4. RNAi-mediated gene expression knockdown

The specific dsRNA primers used in this study were synthesized using an *in vitro* transcription T7 kit (TAKARA, Dalian, China; electronic supplementary material, table S1). dsRNA concentrations were measured using a NanoVue Plus spectrophotometer (GE Healthcare Life Sciences, Little Chalfont, UK). In total, 50 nl of dsRNA of either the target genes or DsRNA-GFP (control) at indicated concentrations (up to 3000 ng μl^−1^) were injected into each worker. Dismemberment assays were performed as above with three technical replicates, and each experiment was repeated at least three times using three different batches of ants. RNAi efficiencies was analysed by qRT-PCR.

### 2.5. Yeast two-hybrid screen

The Matchmaker Gold Yeast Two-Hybrid System (Clontech, USA) was used to screen for SiOBP15 interacting proteins. The complete open reading frame (ORF) of SiOBP15 was cloned into the pGBKT7 plasmid and transformed into the Y2HGold strain. Total RNA from the head and antennae of workers was extracted using TRIzol (Invitrogen, Carlsbad, CA, USA) and reverse transcribed into double-stranded cDNA according to the manufacturer’s protocol. The resulting ds-cDNA library was then cloned into the pGADT7 plasmid and transformed into the Y187 strain. The yeast two-hybrid screen was performed by mixing the bait and prey strains and cultivating them on drop-out media + antibiotics according to the manufacturer’s protocol. Positive clones were isolated, and plasmid inserts were sequenced and analysed via BLAST for identification. Protein–SiOBP15 interactions from the initial screen were confirmed by subsequently cloning the full-length ORFs of select candidates into pGADT7 for one-to-one (individual) Y2H analysis.

### 2.6. Protein expression and surface plasmon resonance

The ORFs corresponding to SiOBP15, SiApoLp-III and SiFABP5 were separately cloned into the pET28a expression vector as His-tag fusion proteins. The integrity of plasmid constructs was verified by sequencing and plasmids were transformed into the *Escherichia coli* BL21 strain for heterologous protein expression. Proteins were purified using a nickel affinity (NI) resin (Ni-Sepharose Cl-6B agarose) according to the manufacturer’s protocol. Purified proteins were analysed by SDS–PAGE for purity and further confirmed by western blotting using an anti-His tag antibody as the probe. His-tags were removed by protease cleavage, and after determining final protein concentrations, aliquots of the purified protein samples were maintained at −80°C. Protein–protein interactions between SiOBP15 and SiApoLp-III and SiOBP15 and SiFABP5 were analysed using open surface plasmon resonance (SPR) by coupling the SiOBP15 protein (ligand) to a COOH chip until saturation. Purified SiFABP5 and SiApoLp-III proteins (analytes) were then added to analyse the kinetics of the interaction between these proteins. Binding kinetics were analysed by titration of the proteins. All experiments were performed three times with the same ‘chip’, and the entire experiment was repeated with three independent COOH-SiOBP15 chip. Dissociation constants were calculated using TraceDrawer (Ridgeview Instruments ab, Sweden) with a one-to-one interaction model.

### 2.7. Glutathione-S-transferase pull-down assays

Constructs (pET32a vector) encoding either glutathione-S-transferase (GST) alone or fusions between GST–SiApoLp-III, GST–SiFABP5 and His–SiOBP15 (constructed above) were expressed in *E. coli* BL21 cells. Proteins were purified using glutathione beads or Ni-Sepharose Cl-6B agarose depending on the fusion partner. For binding/pull-down assays, 1.0−1.5 μg of purified His–SiOBP15 was added to 20 μl of glutathione-agarose to which approximately 2 μg of GST, GST–SiApoLp-III or GST–SiFABP had been added (bound) in 500 μl of binding buffer. Proteins were incubated for 4 h at 4°C. After allowing for protein–protein binding, beads were washed five times in PBS before samples were subjected to SDS–PAGE and imaging.

### 2.8. Fluorescence *in situ* hybridization (FISH)

The antennae of adult worker *S. invicta* were fixed in paraformaldehyde (4%) for 24 h at room temperature, dehydrated in an ethanol series and embedded in LR White resin (Taab, Dermaston, UK). Tissues were sectioned with a microtome (HistoCore Biocut, Leica, Germany) and dried for 2 h at 62°C. Three-colour *in situ* hybridization of the antennal samples was performed using three differentially labelled anti-sense RNA probes (electronic supplementary material, table S1). Anti-sense RNAs corresponding to SiOBP15 (FAM-labelled), SiApoLp-III (cy3-labelled) and SiFABP5 (cy5-labelled) were transcribed from linearized plasmids containing the coding regions of these genes using a T3/T7 RNA transcription system (Roche, China) following the manufacturer’s protocols. Paraffin sections were deparaffinized into water with the following sequence: xylene I for 15 min, xylene II for 15 min, ethanol I for 5 min, ethanol II for 5 min, 85% ethanol for 5 min, 75% ethanol for 5 min and diethyl pyrocarbonate-treated water wash. Sections were boiled in the repair solution for 10−15 min and cooled to room temperature naturally. Proteinase K (20 μg ml^−1^) was added to digest the tissue section at 37°C for 30 min. The sections were rinsed with pure water and washed with PBS three times × 5 min. Pre-hybridization solution was added, incubated at 37°C for 1 h and removed. The hybridization solution containing the probes at a concentration of 8 ng μl^−1^ was then added, and the samples were incubated overnight at 37°C. The hybridization solution was removed and washed sequentially as follows: 2× SSC, 37°C for 10 min; 1× SSC, 37°C for 5 min, 2 times; 0.5× SSC at room temperature for 10 min; 0.5× SSC at room temperature for 10 min. BSA was used as a blocking solution at room temperature for 30 min. The sections were incubated in the dark for 8 min with DAPI staining solution and then rinsed before being sealed with an anti-fluorescence sealer. Sections were observed with an inverted Zeiss Laser Scanning Confocal Microscope (Zeiss LSM 510; Carl Zeiss, Thornwood, NJ, USA)

### 2.9. Protein ligand-binding assays

N-phenyl-1-naphthylamine (1-NPN) was used as a probe for competition (displacement) based fluorescent binding assays as previously described [41]. Samples were analysed using a Hitachi F-2000 fluorescence spectrophotometer at an excitation wavelength of 350 nm and an emission wavelength of 420 nm. The binding constant of the purified proteins to 1-NPN was determined over a substrate concentration range from 2 to 20 μM using 1.0 μM protein in 10 mM HEPES, 100 mM NaCl buffer, pH 7.5. Subsequent competition/displacement assays using test ligands were performed using a concentration of 2 μM 1-NPN, 2 μM protein and 2−40 μM test ligand. Dissociation constants (*K*_*d*_) for 1-NPN were calculated by Scatchard plot analyses using GraphPad Prism 8 Software (GraphPad, La Jolla, CA, USA). The dissociation constants of test ligands (*K*_app_) in 1-NPN competition assays were calculated using the concentration of test ligand required to displace 50% of the 1-NPN signal (IC_50_) values using the following equation: *K*_app_ = (IC50)/(1 + (1 − NPN)/K1 – NPN).

### 2.10. Statistical analysis

Statistical analysis was performed using the software package SPSS v20. qRT-PCR data were analysed by the ΔΔCT methodology. Student’s *t*‐test were used to analyse the significance with two samples. For multiple treatments analysis, one-way ANOVA followed by Bonferroni’s post hoc analysis (equal variance) or Dunnett’s T3 (equal variance not assumed) was used to analyse the significance. A *p* < 0.05 was considered to represent a statistically significant difference.

## 3. Results

### 3.1. Semiochemicals involved in mediating *S. invicta* dismemberment behaviour

**Figure 1.**
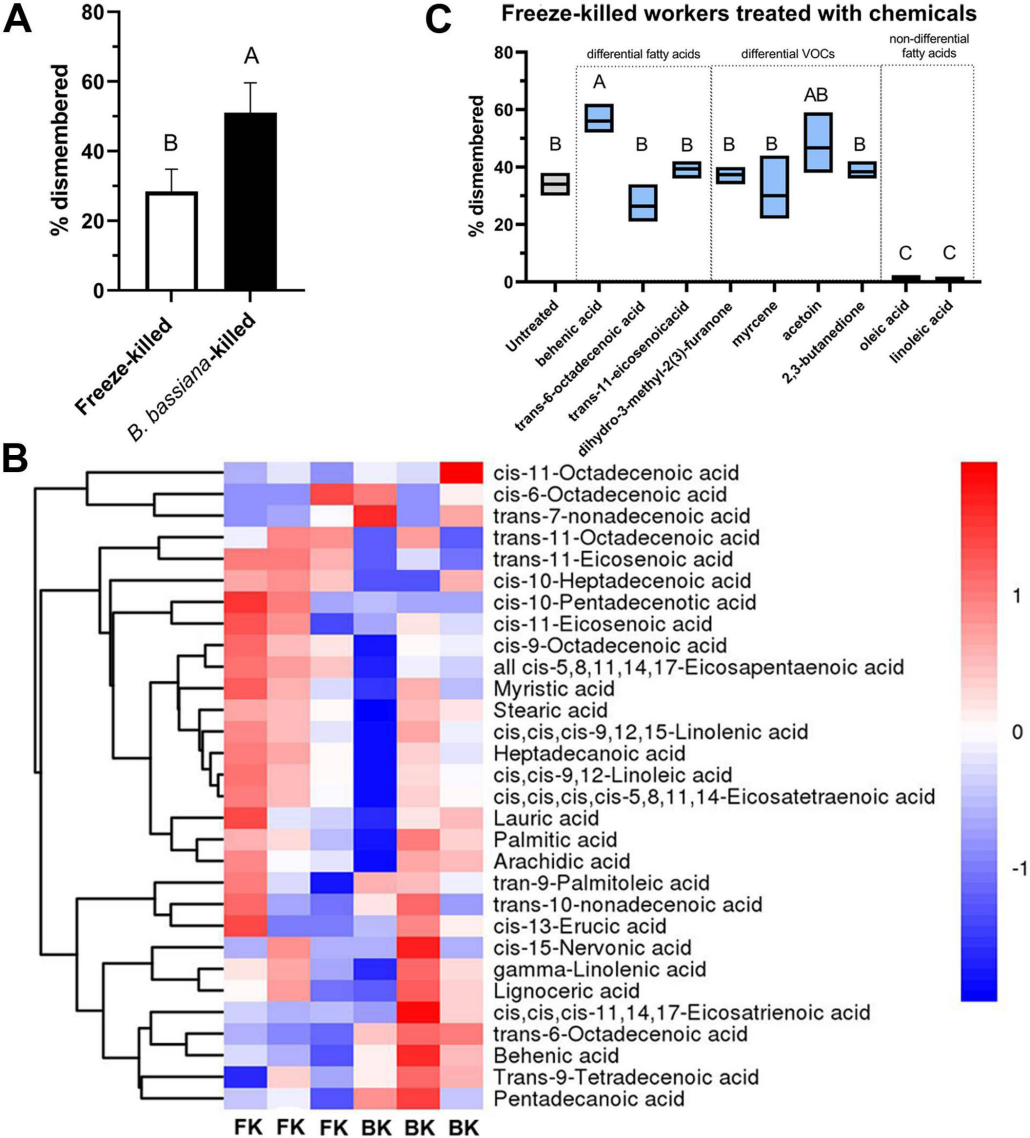
*Solenopsis invicta* social immunity sanitation behaviour: corpse-dismemberment. (*A*) Quantification of corpse-dismemberment by healthy *S. invicta* workers towards freeze-killed and *B. bassiana*-killed conspecifics. (*B*) Heatmap of levels of indicated fatty acids in freeze-killed (CK) and *B. bassiana*-killed (IF) workers quantified by GC–MS. Three independent analyses are shown for each condition. (*C*) Effects of various identified compounds on corpse-dismemberment of freeze-killed conspecifics. All experiments were performed with three independent biological replicates, each consisting of three technical replicates. Error bar = SE. Means followed by different letters are significantly different (Student’s *t*‐test or one-way ANOVA analysis of variance followed by Tukey’s post hoc test, *p* < 0.05).

Healthy *S. invicta* workers exposed to freeze-killed ants engaged in necrophoretic behaviour (corpse removal) to discrete ‘bone piles’ and dismembered those corpses from low to moderate levels (29 ± 6.1%), as defined by severing of limbs, antennae, head and/or abdomen of dead conspecifics (figure 1A; electronic supplementary material, figure S1). However, under identical conditions, when healthy workers were presented with ants killed by *B. bassiana* infection, corpse removal also occurred, but dismemberment levels reached approximately 51 ± 8.7%, representing a 76% increase relative to freeze-killed ants (figure 1A; electronic supplementary material, figure S1; *p* < 0.01).

To identify and quantify potential chemicals that might elicit or inhibit dismemberment behaviour, VOCs from *B. bassiana*-killed and freeze-killed ants were analysed by SPME–GC–MS as detailed in §2. A total of 41 VOCs were found to be significantly differentially present on *B. bassiana*-killed ants compared to freeze-killed ants (electronic supplementary material, figures S2 and S3). In addition, 31 fatty acids were identified from fire ant corpses. The concentrations of trans-6-octadecenoic acid and behenic acid were found to be significantly higher in the *B. bassiana*-killed ants than in freeze-killed ants, whereas the concentration of *trans*-11-eicosenoic acid was significantly higher in freeze-killed ants than in *B. bassiana*-killed ants (figure 1B; electronic supplementary material, figure S4; *p* < 0.01). A panel of seven identified compounds plus oleic and linoleic acids were tested to determine their effects on dismemberment behaviour (figure 1C). Treating freeze-killed ants with behenic acid resulted in a significant increase in dismemberment (*p* < 0.01), whereas treating ants with oleic acid or *cis*,*cis*-9,12-linoleic acid resulted in a significant decrease in dismemberment behaviour (*p* < 0.01) compared to freeze-killed control ants. No significant effects were seen in terms of dismemberment behaviour towards ants treated with *trans*-6-octadecenoic acid, *trans*-11-eicosenoic acid, ihydro-3-methyl-2(3)-furanone, myrcene, acetoin or 2,3-butanedione, as compared to untreated controls.

### 3.2. SiOBP15 contributes to discrimination of corpses killed by *B. bassiana* versus by freezing

**Figure 2.**
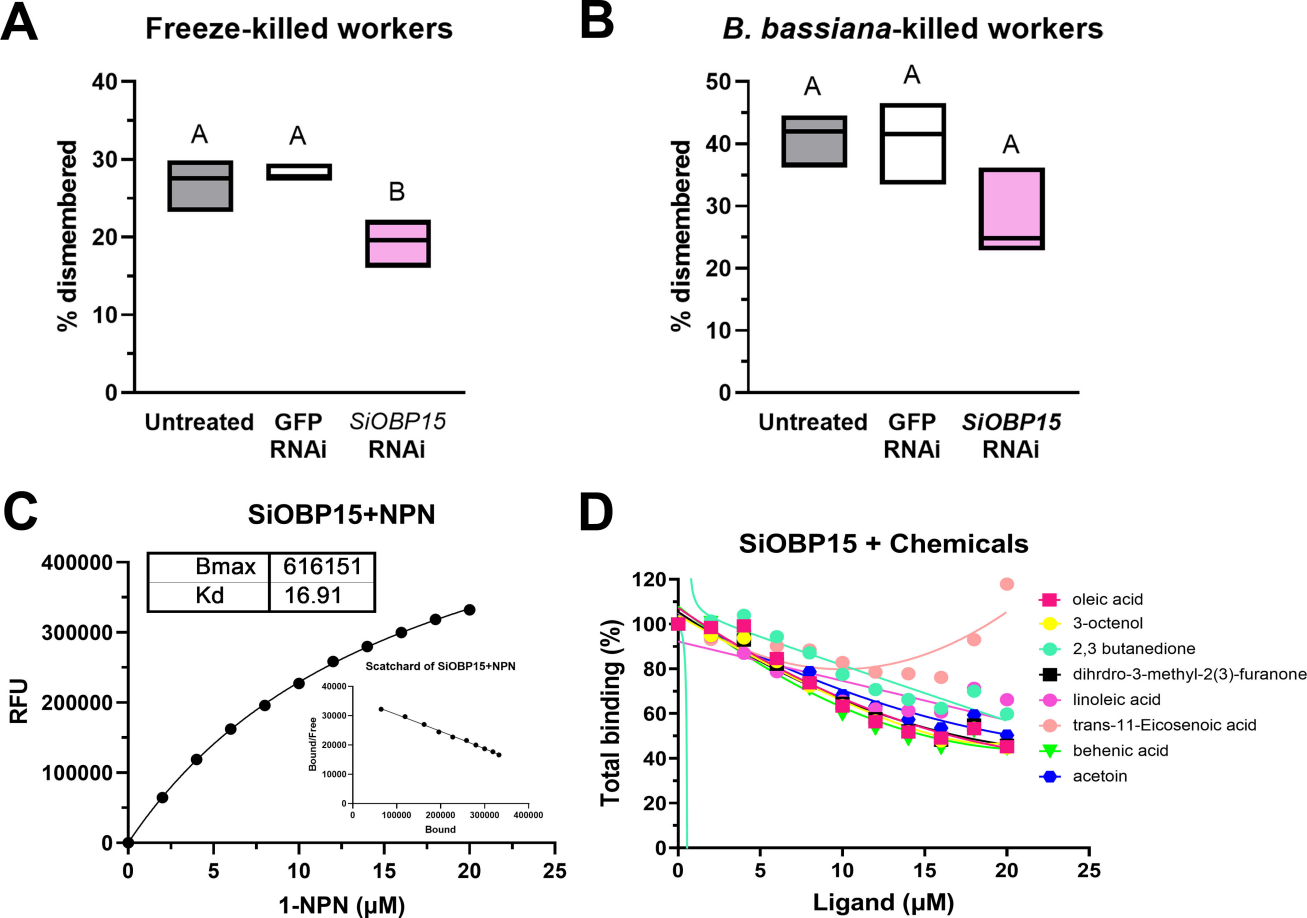
Ligand-binding specificity and contribution of SiOBP15 to corpse-dismemberment. (*A*) Dismemberment (%) of freeze-killed workers by untreated, *GFP*-RNAi and *SiOBP15* RNAi treated workers. (*B*) Dismemberment (%) of *B. bassiana*-killed workers by untreated, *GFP*-RNAi and *SiOBP15* RNAi treated workers. (*C*) Kinetics of binding of purified SiOBP15 to the fluorescent reporter 1-NPN. (*D*) Competition/displacement assays using 1-NPN as the fluorescent reporter ligand bound to purified SiOBP15 using eight identified *S. invicta* VOCs as competing ligands. All experiments were performed with three independent biological replicates, each consisting of three technical replicates. Error bar = SE. Different letter designations indicate significant difference (*p* < 0.05).

SiOBP15 is significantly upregulated between 24 and 72 h after *B. bassiana* infection [42]. To determine functional consequences of SiOBP15 on dismemberment behaviour, RNAi constructs were synthesized and validated for silencing of *SiOBP15* gene expression after microinjection into *S. invicta* workers (electronic supplementary material, figure S5; RNAi-directed towards green fluorescent protein, *gfp*, was used as a control). RNAi-induced knockdown of *SiOBP15* gene expression significantly decreased dismemberment of freeze-killed ants (figure 2A; *p* < 0.01), but surprisingly, no change in dismemberment levels was seen towards *B. bassiana*-killed ants (figure 2B). To characterize the ligand-binding specificity of SiOBP15, we expressed and purified the protein using an *E. coli* heterologous expression system as detailed in §2. We then used the fluorescent reporter substrate 1-NPN (*K*_*d*_ = 16.9 μM) for competition experiments to determine the affinity constants of eight cuticular chemical compounds to purify recombinant SiOBP15 (figure 2C,D). These data showed that linoleic acid, oleic acid, behenic acid, 3-octenol, 2,3 butanedione, acetoin and 3-methyltetrahydro-2-furanone could competitively displace 1-NPN to bind to SiOBP15, with *K*_app_ values of 25.8, 15.46, 14.04, 15.17, 26.52, 26.52 and 16.04 μM, respectively (table 1). No binding to *trans*-11-eicosenoic acid was evident for SiOBP15.

**Table 1.** Binding properties (*K*_app_, competition) of *S. invicta* OBP15, ApoLp-III and FABP5.

chemical	SiOBP15 (μM)	SiOBP15 + SiApoLp III (μM)	SiFABP5 (μM)	SiOBP15 + SiFABP5 (μM)	change on ant cuticle after infection	effect on behaviour
behenic acid	14.04	19.29	13.53	6.75	increases	induces dismemberment
3-octenol	15.17	>500	15.73	12.55	increases	
3-methyltetrahydro-2-furanone	16.04	57.17	16.55	12.81	increases	
*trans*-11-eicosenoic acid	>500	>500	6.56	5.09	decreases	
acetoin	26.52	83.28	16.65	13.73	decreases	
2,3 butanedione	26.52	70.86	16.65	11.85	decreases	
oleic acid	15.46	>500	15.09	10.18	no change	inhibits dismemberment
linoleic acid	25.80	>500	11.46	13.44	no change	inhibits dismemberment

### 3.3. SiApoLp-III and fatty acid binding protein-5 (FABP5) interact with SiOBP15 to mediate corpse-dismemberment behaviour

**Figure 3.**
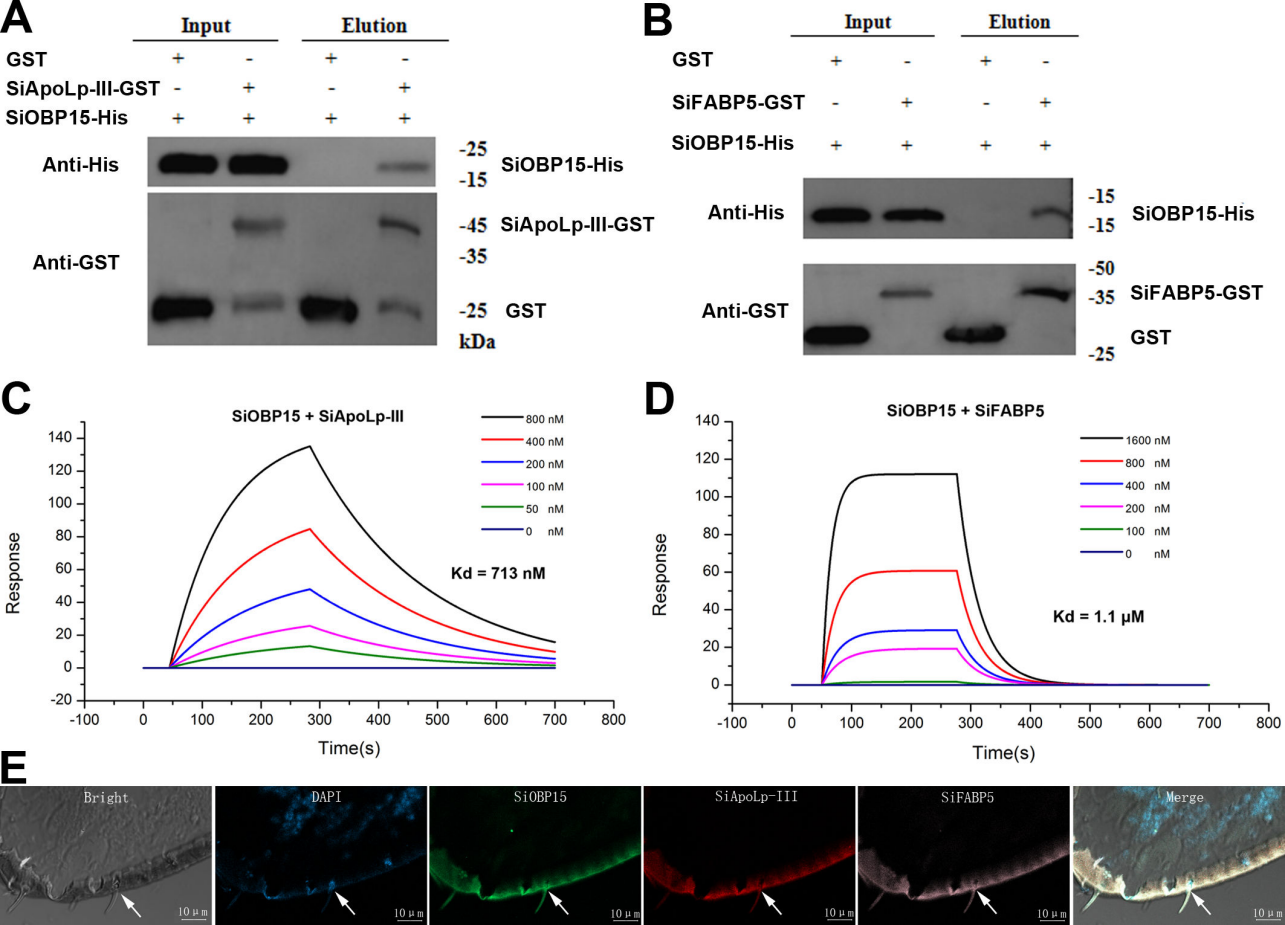
Interactions between SiOBP15 and SiApoLp-III and SiFABP5. Pull-down assays using (*A*) SiOBP15-His and SiApoLp-III-GST and (*B*) SiOBP15-His and SiFABP5-GST. (*C,D*) Purified SiOBP15 was immobilized on chips and used for SPR analyses to determine binding kinetics to SiApoLp-III and SiFABP5, respectively. (*E*) Co-localization of gene expression in *S. invicta* tissues using three-colour FISH. Worker antennae were probed and visualization using anti-sense RNA corresponding to *SiOBP15*, *SiApoLp-III* and *SiFABP5* labelled with green, red and pink fluorescence markers, respectively. Labelling of the same cells by anti-sense *SiOBP15*, *SiApoLp-III* and *SiFABP5* probes are indicated by arrowheads. Co-localization hybridization signals appear as a yellow colour in the overlay (right) of the green, red and pink fluorescence channels. Scale bars = 20 µm.

To identify interacting proteins that contribute to corpse-dismemberment behaviour, a yeast two-hybrid screen in which a SiOBP15 bait construct was screened against an *S. invicta* cDNA expression library using total RNA extracted from head and antennal tissues was performed (electronic supplementary material, figure S6). In total, 16 candidate interacting proteins were identified (electronic supplementary material, table S2) including two (lipid) binding proteins, namely, apolipophorin-III, known to participate in lipid storage/transport and innate immunity, and fatty acid binding protein-5 (SiFABP5), implicated in insect muscle/flight control and immunity. Direct interactions between SiOBP15 and SiApoLp-III or SiFABP5 were confirmed by *in vitro* GST pull-down assays using SiOBP15–His and SiApoLp-III–GST or SiOBP15–His and SiFABP5–GST (figure 3A,B). To further confirm direct association(s) between SiOBP15 and SiApoLp-III and/or SiFABP5, all three proteins were expressed and purified from a recombinant *E. coli* expression system (electronic supplementary material, figure S7), and the kinetics of protein–protein interactions was examined using SPR. Titration of proteins in the SPR experiments and calculation of the dissociation constants revealed that SiOBP15 and SiApoLp-III showed high binding affinity with a dissociation constant (*K*_*d*_) of 713 nM (figure 3C); the SiOBP15–SiFABP5 interaction had a much lower *K*_*d*_ of 1.1 μM (figure 3D). To confirm co-localization of expression, antennal sections were probed with three differentially labelled riboprobes targeted transcripts corresponding to SiOBP15 (green), SiApoLp-III (red) and SiFABP5 (pink; figure 3E).

### 3.4. Interactions between SiOBP15 and SiApoLp-III restrict ligand-binding specificity

Purified SiApoLp-III showed poor binding to the 1-NPN fluorescent reporter probe (calculated *K*_*d*_ = 152 μM, outside the concentration range tested), and hence the probe could not be used to examine substrate binding to this protein (electronic supplementary material, figure S8). However, purified SiFABP5 protein was able to bind 1-NPN, with a dissociation constant (*K*_*d*_) = 11.9 μM (electronic supplementary material, figure S9A). Co-incubation of SiOBP15 with SiApoLp-III indicated that the complex could still be modelled to single 1-NPN binding sites with a dissociation constant of 18.2 μM (electronic supplementary material, figure S9B). Similarly, co-incubation of SiOBP15 with SiFABP5 showed a dissociation constant of approximately 5 μM (electronic supplementary material, figure S9C). Ligand competition experiments showed that SiFABP5 bound all chemicals tested with apparent dissociation constants ranging from *K*_app_ = 6.56 μM for *trans*-11-eicosenoic acid to *K*_app_ = 16.65 μM for acetoin and 2,3 butanedione (electronic supplementary material, figure S9D). Co-incubation of SiOBP15 with SiApoLp-III resulted in a complex with an altered ligand-binding profile depending upon the ligand tested. Binding towards behenic acid remained high, with an apparent dissociation constant of *K*_app_ = 19.29 μM (electronic supplementary material, figure S9E). However, co-incubation of SiOBP15 with SiFABP5 in the ligand-binding assays revealed that the chemical binding range of any complex was similar to that of SiFABP5 alone (electronic supplementary material, figure S9F). SiOBP15 alone was able to bind 3-octenol and oleic and linoleic acids. Incubation of SiOBP15 with SiApoLp-III did not show binding to oleic or linoleic acids (similar to SiApoLp-III alone). In addition, while SiOBP15 alone binds acetoin and 2,3 butanedione, the complex between the two showed limited binding to these compounds. A summary of the binding kinetics data is given in table 1.

### 3.5. Interactions between SiOBP15-SiApoLp-III and ligands induces corpse-dismemberment behaviour, whereas SiOBP15 or SiOBP15-SiFABP5 and ligands inhibits corpse-dismemberment behaviour

**Figure 4.**
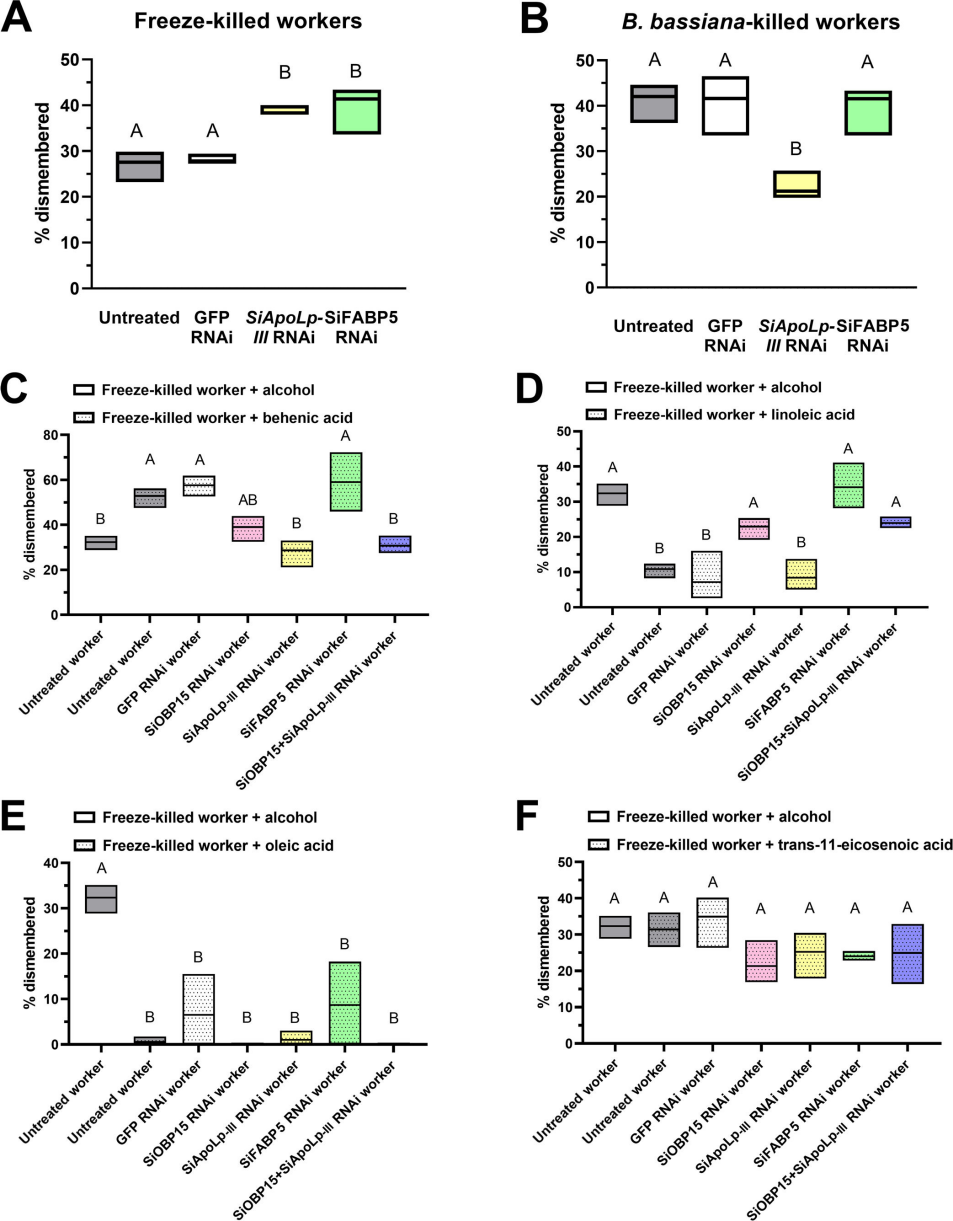
Contributions of SiOBP15, SiApoLp-III and SiFABP5 to dismemberment behaviour. Corpse-dismemberment (%) for untreated controls, and workers treated with RNAi targeting *GFP* (control), *SiApoLp-III* and *SiFABP5* when presented with corpses of workers killed by freezing (*A*) or by *B. bassiana* infection (*B*). Corpse-dismemberment (%) for non-RNAi treated control (WT), and workers treated with RNAi targeting *GFP*, *SiApoLp-III*, *SiFABP5* and both *SiOBP15 + SiApoLp III* (dual RNAi treatment) when presented with freeze-killed workers treated with behenic acid (*C*), linoleic acid (*D*), *trans*-11-eicosenoic acid (*E*) or oleic acid (*F*). All experiments were performed with three independent biological replicates, each consisting of three technical replicates. Error bar = SE. Different letter designations indicate significant difference (*p* < 0.05).

To determine whether SiApoLp-III and SiFABP5 affect dismemberment behaviour, RNAi constructs targeting the expression of these genes were made and validated (electronic supplementary material, figure S5). RNAi-mediated knockdown of *SiApoLp-III* expression resulted in a significant increase in corpse-dismemberment of freeze-killed ants but decreased dismemberment behaviour towards *B. bassiana*-killed ants (*p* < 0.05). *SiFABP5* RNAi-mediated gene expression knockdown also showed a significant increase in corpse-dismemberment of freeze-killed ants (*p* < 0.05) but had no effect on corpse-dismemberment of *B. bassiana*-killed ants (figure 4A,B).

**Figure 5.**
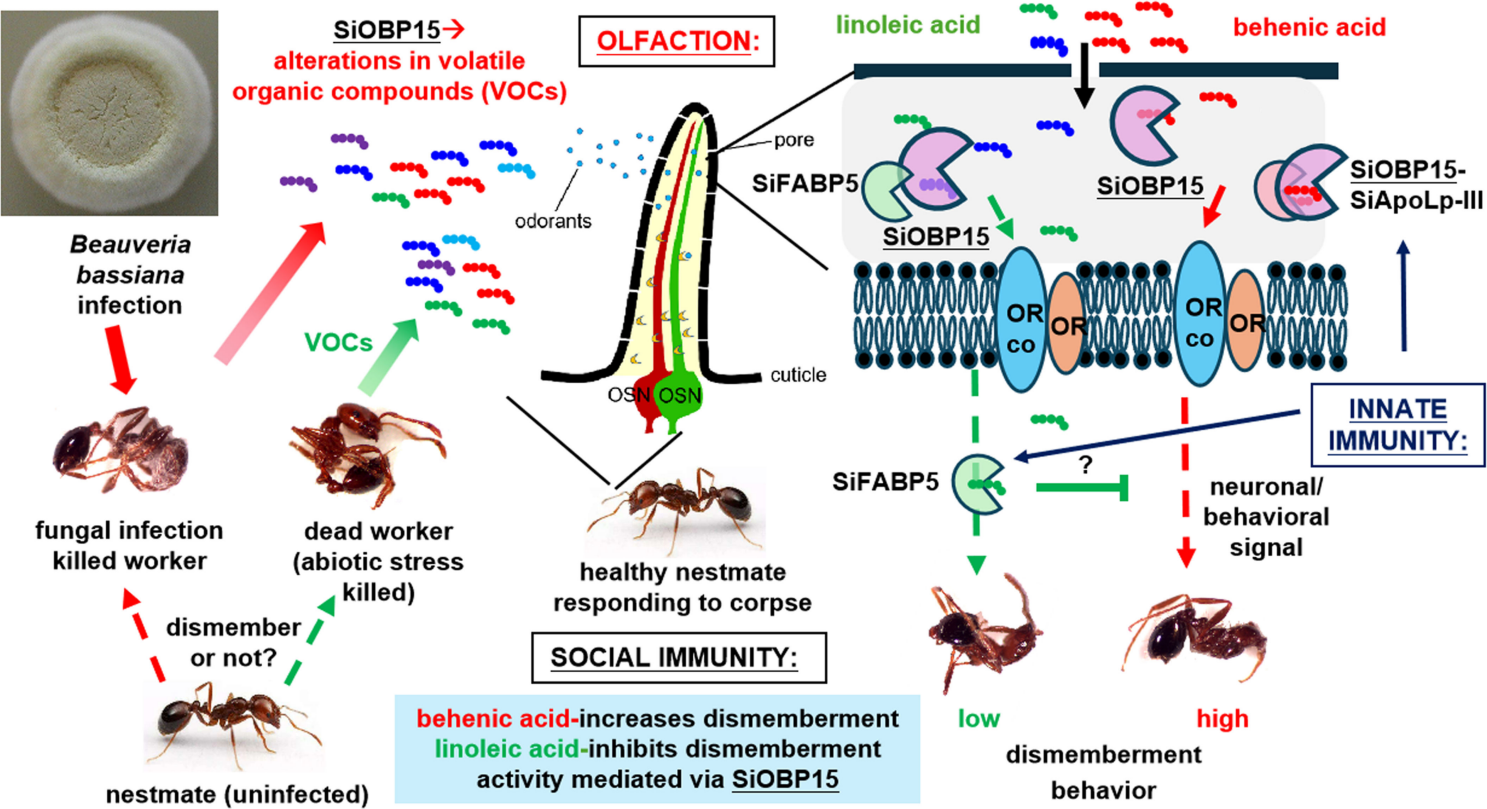
Model for interactions and contributions of *S. invicta* OBP15, ApoLp-III and FABP5 + ligands to corpse-dismemberment sanitation behaviour.

To further confirm the chemical signals recognized by SiOBP15, SiApoLp-III and SiFABP5 and their participation in modulating corpse-dismemberment behaviour in *S. invicta*, the response of workers variously treated with RNAi targeting expression of SiOBP15, SiApoLp-III or SiFABP5 to freeze-killed ants treated with behenic acid, linoleic acid, oleic acid or *trans*-11-eicosenoic acid was investigated. Treatment of freeze-killed workers with behenic acid-induced dismemberment behaviour in both untreated and control (GFP) RNAi workers (figure 4C). RNAi knockdown of SiOBP15, SiApoLp-III or SiOBP15 + SiApoLp III significantly reduced dismemberment behaviour towards freeze-killed workers treated with behenic acid, although targeting of SiOBP15 alone only partially reduced corpse-dismemberment and RNAi targeting SiFABP5 did not reduce dismemberment under these conditions (figure 4C). In contrast, the dismemberment behaviour towards linoleic acid-treated freeze-killed workers was significantly reduced in untreated and *GFP*-RNAi workers compared to control (alcohol-treated) freeze-killed workers (figure 4D; *p* < 0.01). RNAi knockdown of *SiOBP15* increased dismemberment behaviour towards linoleic acid-treated corpses, but below levels seen for the control. In contrast, RNAi *SiApoLp-III* treated workers did not alter the low levels of dismemberment seen towards linoleic acid-treated corpses, whereas RNAi targeting of *SiFABP5* and dual RNAi *SiOBP15 + SiApoLp* III allowed for similar levels of dismemberment of corpses treated with linoleic acid and control corpses (figure 4D). However, although oleic acid treatment of corpses reduced dismemberment behaviour in untreated and *GFP*-RNAi treated workers, RNAi targeting *SiOBP15*, *SiApoLp-III*, *SiFABP5* or *SiOBP15 +SiApoLp* III expression did not restore dismemberment behaviour towards oleic acid-treated freeze-killed workers (figure 4E). In addition, treatment of corpses with *trans*-11-eicosenoic acid did not alter dismemberment behaviour in control or any of the RNAi-targeted treatments (figure 4F). A summary of the effects of RNAi targeting of *SiOBP15*, *SiApo-Lp-III* and *SiFABP5* expression (table 2) and a model of our results are given (figure 5).

**Table 2.** Summary of the effects of RNAi knockdown on corpse dismemberment.

	*RNAi targeting*				
condition	*GFP* RNAi (control)	*SiOBP15*	*SiApoLp-III*	*SiFABP5*	*SiOBP15 + SiApoLp-III*
freeze-killed	(low)	decreased	increased	increased	
*B. bassiana*-killed	(high)	no change	decreased	no change	
freeze-killed + behenic acid	increased^a^	slight decrease^b^	decreased^b^	no change^b^	decreased^b^
freeze-killed + linoleic acid	decreased^a^	increased^c^	no change^c^	increased^c^	increased^c^
freeze-killed + oleic acid	decreased^a^	no change^d^	no change^d^	no change^d^	no change^d^
freeze-killed + eicosenoic acid	no change^a^	no change^e^	no change^e^	no change^e^	no change^e^

^a^ Compared to freeze-killed, no additions.

^b^ Compared to freeze-killed workers treated with behenic acid.

^c^ Compared to freeze-killed workers treated with linoleic acid.

^d^ Compared to freeze-killed workers treated with oleic acid.

^e^ Compared to freeze-killed workers treated with eicosenoic acid.

## 4. Discussion

Social behaviours that include allo-grooming and sanitation are powerful mechanisms to thwart microbial pathogen infection and transmission and can be considered ‘frontline’ defences as they stop infection and/or the spread of the infectious agent into the host before it has occurred. These behaviours are classified as social immunity and involve some aspect of olfaction/chemical sensing and communication to result in behavioural outputs before within-host infection has occurred, whereas innate immune systems act after the pathogen has entered the organism [9]. Both innate immune responses and social immunity (behavioural) responses require specific signals; however, for the latter, such signals, and the molecular mechanisms by which they are decoded and relayed have yet to be described. Many ant species, including *S. invicta*, have evolved complex coordinated behaviours towards dead nestmates, termed sanitation behaviours that help maintain the health of the colony [12]. In cases where colony members die from an infection, sanitation behaviours are thought to reduce the spread of the infection within the colony. One such behaviour exhibited by *S. invicta* in response to *B. bassiana*, a fungal pathogen of insects, is to dismember infected corpses [36]. In other systems, *Lasius niger* queens have also been shown to dismember the corpses of ants exposed to the entomopathogenic fungus, *Metarhizium brunneum*, thus resulting in a higher survival rate [43]. This sanitary behaviour has also been reported in *Zootermopsis angusticollis* [44] and subterranean termites [45], where dismemberment is also thought to inhibit fungal growth, with antimicrobial components released by healthy workers implicated in inhibiting fungal growth on the corpses [46,47]. However, signficant variation can occur in these responses that may be linked to colony and individual member health or other factors.

Several compounds have been shown to either accumulate or be reduced on ant corpses, including linoleic acid and oleic acid that change in fungus-exposed fire ant pupae, and appear to induce corpse removal behaviour [37]. In addition, reduced levels of both dolichodial and iridomyrmecin in dead Argentine ants have been shown to induce corpse removal behaviour [40]. In termites, 3-octanone and 3-octanol are released shortly after death, and these compounds have been shown to induce corpse-dismemberment behaviour [39]. Our data show that in fire ants, behenic acid induces corpse-dismemberment, whereas oleic acid and *cis*, *cis*-9,12-linoleic acid inhibit this behaviour. However, the concentrations of oleic acid and *cis*, *cis*-9,12-linoleic acid did not change in fungus-killed workers compared to freeze-killed workers, suggesting that these chemicals may play a role in guiding the low corpse-dismemberment of freeze-killed nestmates, but not *B. bassiana*-killed workers. In contrast, the concentration of behenic acid was 50% higher in *B. bassiana*-killed workers than in freeze-killed workers, consistent with its activity in inducing dismemberment behaviour in fire ants.

Insects have exquisitely tuned olfactory systems for detection of minute amounts of odorants, e.g. pheromone detection by moths [48]. The peripheral olfactory system of insects includes soluble ligand-binding proteins that deliver odorants to ORs, which trigger neuronal responses that ultimately result in behavioural outcomes [49]. However, demonstrations of direct interactions of binding proteins with ORs are limited, and to our knowledge, direct interactions between OBPs and other soluble ligand-binding proteins have not been reported. We have previously identified of a suite of CSPs and OBPs that were differentially expressed after *B. bassiana* infection of *S. invicta*, with *SiOBP15* identified as among the ones showing the highest levels of change [42]. Here, our data show that SiOBP15 can bind dismemberment-inducing (i.e*.* behenic acid) as well as dismemberment-inhibiting (*cis*, *cis*-9,12-linoleic acid and oleic acid) chemicals. This places SiOBP15 at a pivotal ‘decision’-making crossroad where its activity is modulated by other protein partners. Two important SiOBP15 binding partners, namely SiApoLp-III and SiFABP5, were identified. High affinity between SiOBP15 and SiApoLp-III, with a slightly lower association constant between SiOBP15 and SiFABP5, was seen. These heterocomplexes may represent a hitherto unknown aspect of their functionality. Our results indicate a unique consequence of such interactions summarized as follows: (i) SiOBP15 shows broad substrate specificity, with high affinity to both dismemberment-inducing and dismemberment-inhibiting compounds, (ii) SiApoLp-III alone doesn’t show binding affinity to chemicals, (iii) SiFABP5 shows broad specificity with high affinity to both dismemberment-inducing and inhibiting compounds, (iv) the complex between SiOBP15 and SiApoLp-III results in a shift in substrate specificity, eliminating binding to dismemberment-inhibiting compounds, and (v) SiOBP15 + SiFABP5 results in a complex with high affinity to all compounds tested.

RNAi-mediated knockdown of *SiOBP15* gene expression decreased dismemberment behaviour in general (i.e. when treated workers were exposed to freeze-killed or *B. bassiana*-killed nestmates), but a slight decrease in dismemberment was seen when RNAi-*SiOBP15* treated ants were presented with freeze-killed + behenic acid corpses. A comparison of RNAi-*SiOBP15*-treated ants and RNAi-*GFP*-treated control ants showed that the former dismembered more linoleic acid-treated freeze-killed ants, but not in response to oleic acid- and eicosenoic acid-treated ants. These data indicate that SiOBP15 can act as a mediator to both increase dismemberment behaviour (in response to behenic acid) or to decrease dismemberment behaviour (in response to linoleic acid) but does not seem to contribute to the oleic acid inhibition of dismemberment responses. These data suggest other ligand- binding proteins might mediate the responses to oleic acid. RNAi targeting of *SiApoLp-III* expression increased the dismemberment of freeze-killed ants but decreased this behaviour towards *B. bassiana*-killed ants. These data suggest that one function of SiApoLp-III might be to dampen the dismemberment responses in the absence of fungal cues. However, RNAi-*SiApoLp-III*-treated ants showed decreased responses to behenic acid-treated corpses, indicating that SiApoLp-III also positively contributes to dismemberment in the presence of the appropriate ligand. By itself, SiApoLp-III did not appear to be significantly involved in responses to either oleic or linoleic acid. RNAi-mediated knockdown of both *SiOBP15* and *SiApoLp-III* expression resulted in a somewhat more pronounced phenotype compared to RNAi-*SiOBP15* treatment alone. These results suggest that SiOBP15–SiApoLp-III complex may function cooperatively to respond to behenic acid in the dismemberment-inducing pathway. RNAi knockdown of *SiFABP5* expression had no effects on dismemberment of either *B. bassiana*-killed ants or behenic acid- or oleic acid-treated ants, suggesting that SiFABP5 acts to inhibit dismemberment in the presence of linoleic acid.

## 5. Conclusions

Our data define a circuit involving at least three ligand-binding proteins, namely SiBOP15, SiApoLp-III and SiFABP5. These proteins link olfaction-related proteins/processes to members of the lipoprotein family implicated in immunity and/or muscle control/neural development. The consequences of these interactions help to regulate the degree to which (social immunity) sanitation behaviour, namely dismemberment, is engaged by healthy workers towards nestmates killed by either abiotic stress (freezing) or by fungal infection. We show that an apolipophorin and a fatty acid binding protein were capable of forming heterocomplexes with an odorant binding protein. Each of these proteins, on their own, have been implicated in a wider nexus of physiological processes from muscle/flight activity to immunity, opening up the possibility that these processes can be impacted by OBPs (SiOBP15 in particular) and the various ligands identified. Our data establish a molecular connection in which an OBP bridges olfaction to canonical immunity and then to social immunity behaviours. These findings expand our understanding of host–pathogen behavioural and innate immune interactions to include processes connecting olfactory OBPs to apolipophorins and other lipid transport proteins involved in innate immunity and other processes to behavioural outputs.

## Data Availability

All data are included in the main manuscript and/or supplemental materials, which are available online [50].

## References

[1] Plowes N. 2010 An introduction to eusociality. Nat. Educ. Knowl **3**, 7.

[2] Nowak MA, Tarnita CE, Wilson EO. 2010 The evolution of eusociality. Nature **466**, 1057–1062. (10.1038/nature09205) 20740005 PMC3279739

[3] Yan H, Simola DF, Bonasio R, Liebig J, Berger SL, Reinberg D. 2014 Eusocial insects as emerging models for behavioural epigenetics. Nat. Rev. Genet. **15**, 677–688. (10.1038/nrg3787) 25200663

[4] Pereira RM, Stimac JL. 1992 Transmission of Beauveria bassiana within Nests of Solenopsis invicta (Hymenoptera: Formicidae) in the Laboratory. Environ. Entomol. **21**, 1427–1432. (10.1093/ee/21.6.1427)

[5] Schmid-Hempel P. 1998 Parasites in social insects. Princeton, NJ: Princeton University Press. (10.1515/9780691206851)

[6] Zhang W, Tettamanti G, Bassal T, Heryanto C, Eleftherianos I. 2021 Regulators and signalling in insect antimicrobial innate immunity: Functional molecules and cellular pathways. Cell. Signal. **83**. (10.1016/j.cellsig.2021.110003) 33836260

[7] Liu L, Zhao XY, Tang QB, Lei CL, Huang QY. 2019 The mechanisms of social immunity against fungal infections in eusocial insects. Toxins **11**, 244. (10.3390/toxins11050244) 31035652 PMC6563085

[8] Meunier J. 2015 Social immunity and the evolution of group living in insects. Phil. Trans. R. Soc. B **370**, 20140102. (10.1098/rstb.2014.0102) 25870389 PMC4410369

[9] Cremer S. 2019 Social immunity in insects. Curr. Biol. **29**, R458–R463. (10.1016/j.cub.2019.03.035) 31163158

[10] Qiu H long, Lu L hua, Shi Q xing, He Y rong. 2014 Fungus exposed Solenopsis invicta ants benefit from grooming. J. Insect Behav. **27**, 678–691. (10.1007/s10905-014-9459-z)

[11] Sun Q, Zhou X. 2013 Corpse management in social insects. Int. J. Biol. Sci. **9**, 313–321. (10.7150/ijbs.5781) 23569436 PMC3619097

[12] Howard DF, Tschinkel WR. 1976 Aspects of necrophoric behavior in the red imported fire ant, Solenopsis invicta. Behaviour **56**, 157–178. (10.1163/156853976x00334)

[13] Ai S, Zhang Y, Chen Y, Zhang T, Zhong G, Yi X. 2022 Insect-microorganism interaction has implicates on insect olfactory systems. Insects **13**, 1094. (10.3390/insects13121094) 36555004 PMC9787996

[14] Miazzi F, Jain K, Kaltofen S, Bello JE, Hansson BS, Wicher D. 2022 Targeting insect olfaction in vivo and in vitro using functional imaging. Front. Cell. Neurosci. **16**, 839811. (10.3389/fncel.2022.839811) 35281299 PMC8907589

[15] Pyzza PB, Newhall KA, Kovačič G, Zhou D, Cai D. 2021 Network mechanism for insect olfaction. Cogn. Neurodynamics **15**, 103–129. (10.1007/s11571-020-09640-3) PMC7947141 33786083

[16] Sato K, Touhara K. 2008 Insect olfaction: receptors, signal transduction, and behavior. Results Probl. Cell Differ **47**, 203–220. (10.1007/400_2008_10) 19083129

[17] Rützler M, Zwiebel LJ. 2005 Molecular biology of insect olfaction: recent progress and conceptual models. J. Comp. Physiol. A Neuroethol. Sens. Neural. Behav. Physiol. **191**, 777–790. (10.1007/s00359-005-0044-y) 16094545

[18] Jacquin-joly E, Merlin C. 2004 Insect olfactory receptors: contributions of molecular biology to chemical ecology. J. Chem. Ecol. **30**, 2359–2397. (10.1007/s10886-004-7941-3) 15724962

[19] Leal WS. 2013 Odorant reception in insects: roles of receptors, binding proteins, and degrading enzymes. Annu. Rev. Entomol. **58**, 373–391. (10.1146/annurev-ento-120811-153635) 23020622

[20] Zhang W *et al*. 2023 An odorant binding protein is involved in counteracting detection-avoidance and Toll-pathway innate immunity. J. Adv. Res. **48**, 1–16. (10.1016/j.jare.2022.08.013) 36064181 PMC10248801

[21] Zhang J, Mao K, Ren Z, Jin R, Zhang Y, Cai T, He S, Li J, Wan H. 2022 Odorant binding protein 3 is associated with nitenpyram and sulfoxaflor resistance in Nilaparvata lugens. Int. J. Biol. Macromol. **209**, 1352–1358. (10.1016/j.ijbiomac.2022.04.100) 35460755

[22] Zhang Y *et al*. 2023 SmCSP4 from aphid saliva stimulates salicylic acid‐mediated defence responses in wheat by interacting with transcription factor TaWKRY76. Plant Biotechnol. J. **21**, 2389–2407. (10.1111/pbi.14139) 37540474 PMC10579719

[23] Li S, Picimbon JF, Ji S, Kan Y, Chuanling Q, Zhou JJ, Pelosi P. 2008 Multiple functions of an odorant-binding protein in the mosquito Aedes aegypti. Biochem. Biophys. Res. Commun. **372**, 464–468. (10.1016/j.bbrc.2008.05.064) 18502197

[24] Li ZQ, Zhang S, Cai XM, Luo JY, Dong SL, Cui JJ, Chen ZM. 2018 Distinct binding affinities of odorant-binding proteins from the natural predator Chrysoperla sinica suggest different strategies to hunt prey. J. Insect Physiol. **111**, 25–31. (10.1016/j.jinsphys.2018.10.004) 30336148

[25] He P, Chen GL, Li S, Wang J, Ma YF *et al*. 2019 Evolution and functional analysis of odorant-binding proteins in three rice planthoppers: Nilaparvata lugens, Sogatella furcifera, and Laodelphax striatellus. Pest Manag. Sci. **75**, 1606–1620. (10.1002/ps.5277) 30515974

[26] Li K, Wang S, Zhang K, Ren L, Ali A, Zhang Y, Zhou J, Guo Y. 2014 Odorant binding characteristics of three recombinant odorant binding proteins in Microplitis mediator (Hymenoptera: Braconidae). J. Chem. Ecol. **40**, 541–548. (10.1007/s10886-014-0458-5) 24928754

[27] Ross KG. 2002 Identification of a major gene regulating complex social behavior. Science **295**, 328–332. (10.1126/science.1065247) 11711637

[28] Guarna MM *et al*. 2015 A search for protein biomarkers links olfactory signal transduction to social immunity. BMC Genom. **16**, 63. (10.1186/s12864-014-1193-6) PMC4342888 25757461

[29] Zheng R, Xie M, Keyhani NO, Xia Y. 2023 An insect chemosensory protein facilitates locust avoidance to fungal pathogens via recognition of fungal volatiles. Int. J. Biol. Macromol. **253**, 127389. (10.1016/j.ijbiomac.2023.127389) 37827395

[30] Zhang W, Chen X, Eleftherianos I, Mohamed A, Bastin A, Keyhani NO. 2024 Cross-talk between immunity and behavior: insights from entomopathogenic fungi and their insect hosts. FEMS Microbiol. Rev. **48**. (10.1093/femsre/fuae003) PMC10883697 38341280

[31] Ascunce MS, Yang CC, Oakey J, Calcaterra L, Wu WJ, Shih CJ, Goudet J, Ross KG, Shoemaker D. 2011 Global invasion history of the fire ant Solenopsis invicta. Science **331**, 1066–1068. (10.1126/science.1198734) 21350177

[32] Menchetti M, Schifani E, Alicata A, Cardador L, Sbrega E, Toro-Delgado E, Vila R. 2023 The invasive ant Solenopsis invicta is established in Europe. Curr. Biol. **33**, R896–R897. (10.1016/j.cub.2023.07.036) 37699343

[33] Martinez-Ruiz C, Pracana R, Stolle E, Paris CI, Nichols RA, Wurm Y. 2020 Genomic architecture and evolutionary antagonism drive allelic expression bias in the social supergene of red fire ants. eLife **9**, e55862. (10.7554/elife.55862) 32773032 PMC7476760

[34] Chen J, Zhou Y, Lei Y, Shi Q, Qi G, He Y, Lyu L. 2022 Role of the foraging gene in worker behavioral transition in the red imported fire ant, Solenopsis invicta (Hymenoptera: Formicidae). Pest Manag. Sci. **78**, 2964–2975. (10.1002/ps.6921) 35419943

[35] Siebeneicher SR, Kenerley CM. 1992 Infection of the red imported fire ant by Beauveria bassiana through various routes of exposure. J. Invertebr. Pathol. **59**, 280–285. (10.1016/0022-2011(92)90133-O)

[36] Qiu HL, Qin CS, Fox EGP, Wang DS, He YR. 2020 Differential behavioral responses of Solenopsis invicta (Hymenoptera: Formicidae) workers toward nestmate and non-nestmate corpses. J. Insect Sci. **20**, 11. (10.1093/jisesa/ieaa069) PMC7387867 32725158

[37] Qiu HL, Lu LH, Shi QX, Tu CC, Lin T, He YR. 2015 Differential necrophoric behaviour of the ant Solenopsis invicta towards fungal-infected corpses of workers and pupae. Bull. Entomol. Res. **105**, 607–614. (10.1017/s0007485315000528) 26082426

[38] Swanson JAI, Torto B, Kells SA, Mesce KA, Tumlinson JH, Spivak M. 2009 Odorants that induce hygienic behavior in honeybees: identification of volatile compounds in chalkbrood-infected honeybee larvae. J. Chem. Ecol. **35**, 1108–1116. (10.1007/s10886-009-9683-8) 19816752

[39] Sun Q, Haynes KF, Zhou X. 2017 Dynamic changes in death cues modulate risks and rewards of corpse management in a social insect. Funct. Ecol. **31**, 697–706. (10.1111/1365-2435.12754)

[40] Choe DH, Millar JG, Rust MK. 2009 Chemical signals associated with life inhibit necrophoresis in Argentine ants. Proc. Natl Acad. Sci. USA **106**, 8251–8255. (10.1073/pnas.0901270106) 19416815 PMC2688878

[41] Li HL, Song XM, Wu F, Qiu YL, Fu XB, Zhang LY, Tan J. 2020 Chemical structure of semiochemicals and key binding sites together determine the olfactory functional modes of odorant-binding protein 2 in Eastern honey bee, Apis cerana. Int. J. Biol. Macromol. **145**, 876–884. (10.1016/j.ijbiomac.2019.11.189) 31765753

[42] Wei Z, Ortiz-Urquiza A, Keyhani NO. 2021 Altered expression of chemosensory and odorant binding proteins in response to fungal infection in the red imported fire ant, Solenopsis invicta. Front. Physiol. **12**, 596571. (10.3389/fphys.2021.596571) 33746766 PMC7970113

[43] Pull CD, Cremer S. 2017 Co-founding ant queens prevent disease by performing prophylactic undertaking behaviour. BMC Evol. Biol. **17**, 219. (10.1186/s12862-017-1062-4) 29025392 PMC5639488

[44] Rosengaus R, Traniello JF. 2001 Disease susceptibility and the adaptive nature of colony demography in the dampwood termite Zootermopsis angusticollis. Behav. Ecol. Sociobiol. **50**, 546–556. (10.1007/s002650100394)

[45] Chouvenc T, Su NY. 2012 When subterranean termites challenge the rules of fungal epizootics. PLoS One **7**, e34484. (10.1371/journal.pone.0034484) 22470575 PMC3314638

[46] Storey GK, Vander Meer RK, Boucias DG, McCoy CW. 1991 Effect of fire ant (Solenopsis invicta) venom alkaloids on the in vitro germination and development of selected entomogenous fungi. J. Invertebr. Pathol. **58**, 88–95. (10.1016/0022-2011(91)90166-n)

[47] Kesäniemi J, Koskimäki JJ, Jurvansuu J. 2019 Corpse management of the invasive Argentine ant inhibits growth of pathogenic fungi. Sci. Rep. **9**, 9. (10.1038/s41598-019-44144-z) 31110201 PMC6527551

[48] Ishikawa Y. 2020 Insect sex pheromone research and beyond: from molecules to robots. Singapore: Springer. (10.1007/978-981-15-3082-1)

[49] Hansson BS, Stensmyr MC. 2011 Evolution of insect olfaction. Neuron **72**, 698–711. (10.1016/j.neuron.2011.11.003) 22153368

[50] Zhang W, Chen X, Tian J, Schal C, Mohamed A, Zang L *et al*. 2025 Supplementary material from: An odorant binding protein functions in fire ant social immunity, interfacing with innate immunity. Figshare (10.6084/m9.figshare.c.7653691)39933575

